# Risk assessment and prevention in airport security assurance by integrating LSTM algorithm

**DOI:** 10.1371/journal.pone.0315799

**Published:** 2025-01-03

**Authors:** Yao Hu, Liguang Qiao, Feng Gu

**Affiliations:** 1 College of Civil Aviation Safety Engineering, Civil Aviation Flight University of China, Guanghan, Sichuan, China; 2 College of Economics and Management, Civil Aviation Flight University of China, Guanghan, Sichuan, China; 3 Logistics service company, Civil Aviation Flight University of China, Guanghan, Sichuan, China; Ataturk University, TÜRKIYE

## Abstract

The risk assessment and prevention in traditional airport safety assurance usually rely on human experience for analysis, and there are problems such as heavy manual workload, excessive subjectivity, and significant limitations. This article proposed a risk assessment and prevention mechanism for airport security assurance that integrated LSTM algorithm. It analyzed the causes of malfunctioning flights by collecting airport flight safety log datasets. This article extracted features related to risk assessment, such as weather factors, airport facility inspections, and security check results, and conducted qualitative and quantitative analysis on these features to generate a datable risk warning weight table. This article used these data to establish an LSTM model, which trained LSTM to identify potential risks and provide early warning by learning patterns and trends in historical data. It then handed over the new data to the trained LSTM model for risk assessment and prediction, grading and warning of risks. It monitored the airport security situation in real-time based on the results and quickly notified airport security personnel to handle it. The outcome indicates that the standard error of the LSTM algorithm model training is less than 0.18, and the decision coefficients were all greater than 0.9. The predicted data was highly consistent with the actual data. It can be summarized that the algorithmic model has good accuracy and robustness. The LSTM algorithm can play a role in providing early warning, assisting decision-making, optimizing resources, and enhancing real-time monitoring in airport security assurance. It can effectively improve the safety and prevention capabilities of airports, and reduce the losses caused by potential risks.

## 1. Introduction

With the rapid growth of China’s economy, the demand for civil aviation transportation has also experienced rapid growth. As a hub for air transportation, increasingly renowned airports bear a large number of flight transportation responsibilities around the world. Tens of thousands of planes carry passengers and goods to and from airports. At this time, there may be safety hazards in the takeoff and landing, ground handling, and security checks of the aircraft. The occurrence of safety incidents at the airport may lead to serious consequences such as facility damage, flight delays, economic losses, and casualties. Currently, with the increase in global terrorism, illegal trafficking, and other criminal activities, airport security is facing various security threats. These threats include terrorist attacks, hijacking, illegal goods transportation, etc. These threats not only affect airport operations, but also involve national and public security aspects. Many experts also express the importance of building an efficient and comprehensive national air transportation system [1].

In order to ensure airport safety, various countries have formulated a series of regulations and regulatory requirements, covering safety measures, emergency response plans, risk assessment, and other aspects. Risk assessment of airports can help airport managers and relevant departments understand the risk situation and take corresponding measures to reduce and manage risks. Majid S and others [2] used quantitative methods for safety risk management and the impact of airport personnel capabilities on airport flight safety performance. This study involved airport staff and up to 60 officials interviewed. The results indicate that safety risk management has a significant direct impact on flight safety performance. Eckhart M and others [3] believed that risk assessment was a component of identifying, analyzing, and evaluating risks in the risk management process. In this context, quantitative evaluation is crucial. Qualitative and quantitative assessments can be conducted based on risk factors, degree of danger, and vulnerability concepts, or qualitative and quantitative analysis can be conducted on some representative events [4–6]. Scholars have proposed that multiple dimensions should be considered in risk assessment, and they believe that comprehensive consideration of different dimensions of risk can provide a more comprehensive assessment of airport safety and security. Kelemen M and others [7] proposed a fuzzy expert model based on aviation practice. The chosen scenarios and entered data can be used as an educational message model and software for risk assessment of airport networks and information systems. It is mainly used for education and to train professionals in aviation to ensure safe and sustainable development of air transportation. Kaewunruen S [8] proposed four strategies to minimize losses by rigorously evaluating potential losses under various threat scenarios. He screened passengers, monitors systems, improved cargo screening rates, and explosion-proof containers through observation technology. When the value of security measures equals their cost, cost and efficiency assessments are used to determine the most suitable security measures. Janssen S and others [9] introduced a new agent based modeling and simulation method that uses formal social technology models (including time and space aspects) to perform security risk management for airport operations. Khan N and others [10] evaluated the biometric exit procedures. These methods can enhance airport security, but current risk assessment relies too heavily on subjective judgment based on manual experience. It usually has inaccuracies, and human resources are often unable to handle the correlations and changing trends between large amounts of data, which limits the ability to prevent and mitigate risks. The situation at each airport is different, and the assessment cannot be universally applicable, resulting in the need to invest more resources in analysis and processing, which limits its application and efficiency in the security guarantee of large airports.

In order to strengthen airport security, big data analysis and artificial intelligence technology can be applied, and machine learning (ML) and data mining can be used to achieve data processing. Janiesch C and others [11] believed that algorithms based on ML and deep learning (DL) were emerging methods for solving time series prediction problems. These techniques have been shown to yield more accurate outcomes compared to classic regression modeling. Smagulova K [12] found that Recurrent Neural Network (RNN) is an effective tool. It is used to approximate dynamic systems and process time and sequence related data, such as video, audio, and others. Staudemeyer R C [13] believed that the Long Short-Term Memory RNN (LSTM-RNN) was currently one of the most powerful dynamic classifiers known. LSTM is a RNN with state memory and multi-layer cellular structure. As a special kind of RNN, LSTM resolves the gradient vanishing and gradient explosion problems of traditional RNNs when dealing with long sequence data by introducing a gating mechanism. Yu Y and others [14] can effectively handle long-term dependency problems by introducing entry-level functions into unit structures. Since its inception, almost all exciting results based on RNN have been achieved by LSTM. LSTM has become a research hotspot in DL. Due to its effectiveness in a wide range of practical applications, the LSTM network has been widely reported in scientific journals, technology blogs, and implementation guides [15]. On the basis of demonstrating its robustness and higher prediction accuracy, Shahid F [16] believed that LSTM can be used for certain risk prediction to better plan and manage monitoring of these risks. The LSTM algorithm can play a good auxiliary role in processing airport safety data sorted by long-term time series.

The study goal of this article is to develop an innovative airport security risk assessment and prevention mechanism, which improves the accuracy and efficiency of airport security by integrating Long Short-Term Memory (LSTM) algorithm. The research methodology consists of the following steps: First, key features related to risk assessment are identified and extracted by collecting and analyzing the airport flight safety log dataset. Then, these characteristics are qualitatively and quantitatively analyzed to generate a quantifiable risk warning weight table. This data is then used to build an LSTM model, which identifies potential risks and provides early warnings by learning patterns and trends in historical data. Finally, the new data was input into the trained LSTM model for real-time risk assessment, prediction, classification and early warning, and the airport security personnel were notified in time for processing. By combining the LSTM algorithm with airport security risk assessment, this paper realizes the automatic processing of the whole chain from data collection, feature extraction, model training to risk prediction and early warning. Experiments show that the model is accurate and robust, which can effectively improve the efficiency of risk assessment, provide decision support for airport managers, optimize resource allocation, enhance monitoring capabilities, and improve the overall security and prevention level of the airport.

Article contributions:

1. This article applies the LSTM deep learning model to the field of airport security for the first time, breaking through the limitations of traditional static risk assessment. The experiment is based on historical flight data and related fault characteristics. The LSTM model dynamically predicts potential safety risks, provides more accurate risk warnings, and innovatively improves the real-time and predictive capabilities of airport risk assessment.
2. Research and design an exclusive feature extraction method for airport safety, explore key influencing factors such as weather changes, flight delays, and failure frequency, and optimize the risk assessment index system to make it more applicable and applicable in the field of airport safety. Robustness is significantly improved.
3. The experiment uses an LSTM-based intelligent early warning system framework, which is suitable for airport security and has good scalability. The system can update data in real time, automatically learn new risk models, and has adaptive capabilities to ensure its stable performance in different complex environments.

## 2. Methods

### 2.1 Clarifying risk elements

**Fig 1:**
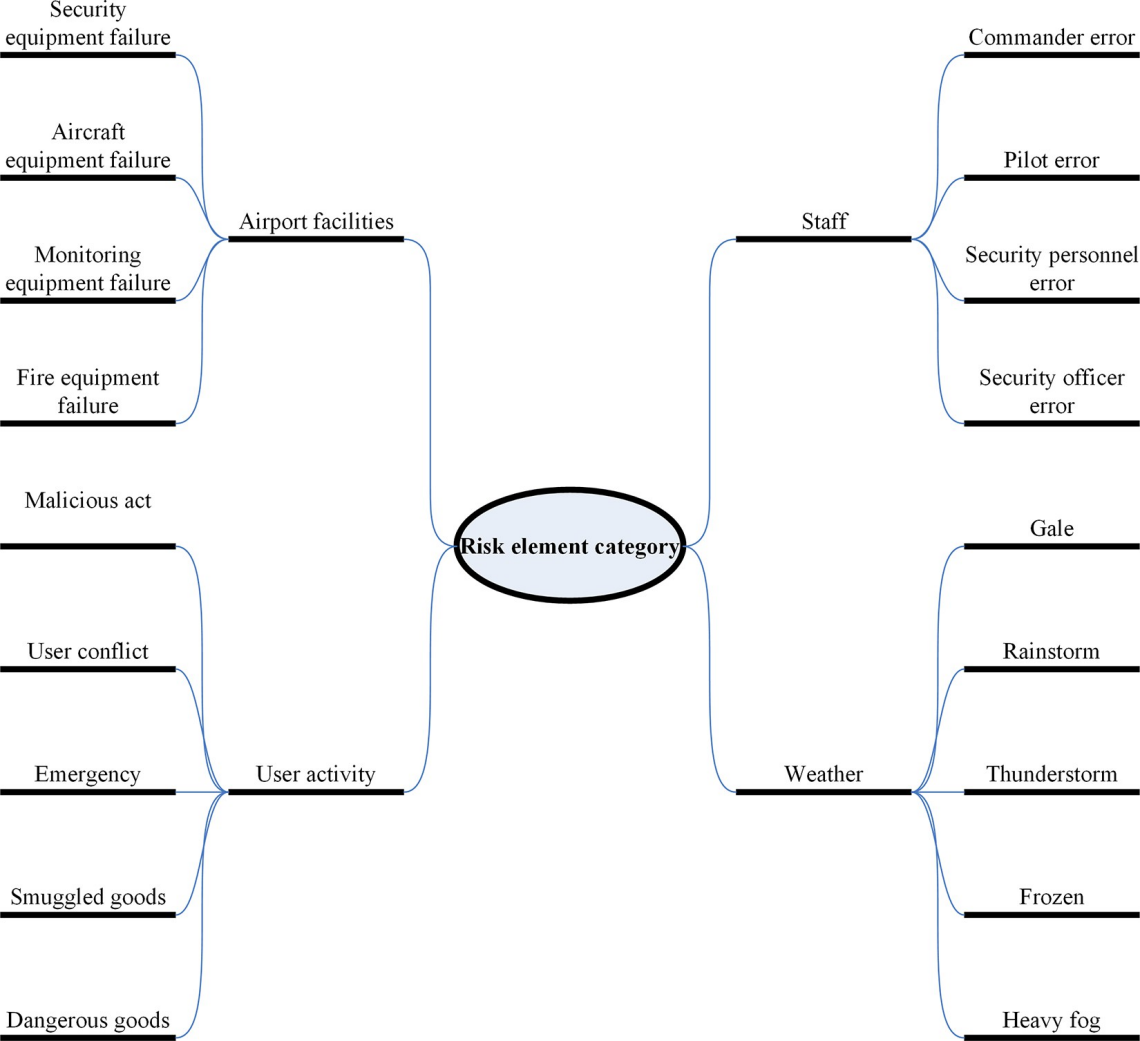
Risk element category diagram.

The first step in implementing model algorithms is to identify risk factors. Firstly, airport safety assurance carries out classification risk identification to achieve analysis of risk factors in different aspects, and on this basis, classifies influential risk factors. It analyzes the characteristics of flight delays, qualitatively analyzes the varying degrees of correlation of flight delays under various influencing conditions, and combines with the normal statistical methods of civil aviation flights. A single flight failure table can be created for airlines, airports, weather, and other aspects [17], as shown in Table 1. Among these reasons, there are some risk factors with high contingency or weak correlation, such as air traffic control system failures, sudden user diseases, etc. If this factor is involved in model training, it would make model training more complex and reduce accuracy. In order to simplify model training, this article selects some risk factors with strong correlations. Wang Yonggang and others [18] proposed the use of fault tree analysis to identify safety risk factors. He used grey correlation analysis and expert scoring to systematically analyze safety risks and obtain the risk values of each factor. He proposed risk management strategies for factors with high risk values. Based on a large amount of historical data accumulation, expert experience in various fields, and investigation and analysis by grassroots units, this article integrates the risk factors of airport safety assurance into four major categories after evaluation (Fig 1).

**Table 1:** Flight failure factors.

Weather	Mechanical equipment	Airport facilities	Staff error	User activity	Other
					Information
	Failure				
Gale	Engine	Security equipment failure	Pilot error	Carrying dangerous	Air traffic control system failure
	Equipment				
				Items	
	Failure				
Thunderstorm	Landing	Monitoring equipment failure	Security officer error	Smuggled goods	Special event
	Gear				
	Failure				
Rainstorm	Electrical equipment	Fire equipment failure	Security personnel error	User conflict	
	Failure				
Frozen	Navigation equipment	Infrastructure failure	Commander	Emergency	
			Error		
	Failure				
Heavy fog				Malicious act	

**Fig 1** lists various risk factors that may lead to adverse outcomes and classifies them into different categories: Airport facilities, Staff, User activity and Weather.

### 2.2 Establishing intelligent risk assessment model

**Fig 2:**
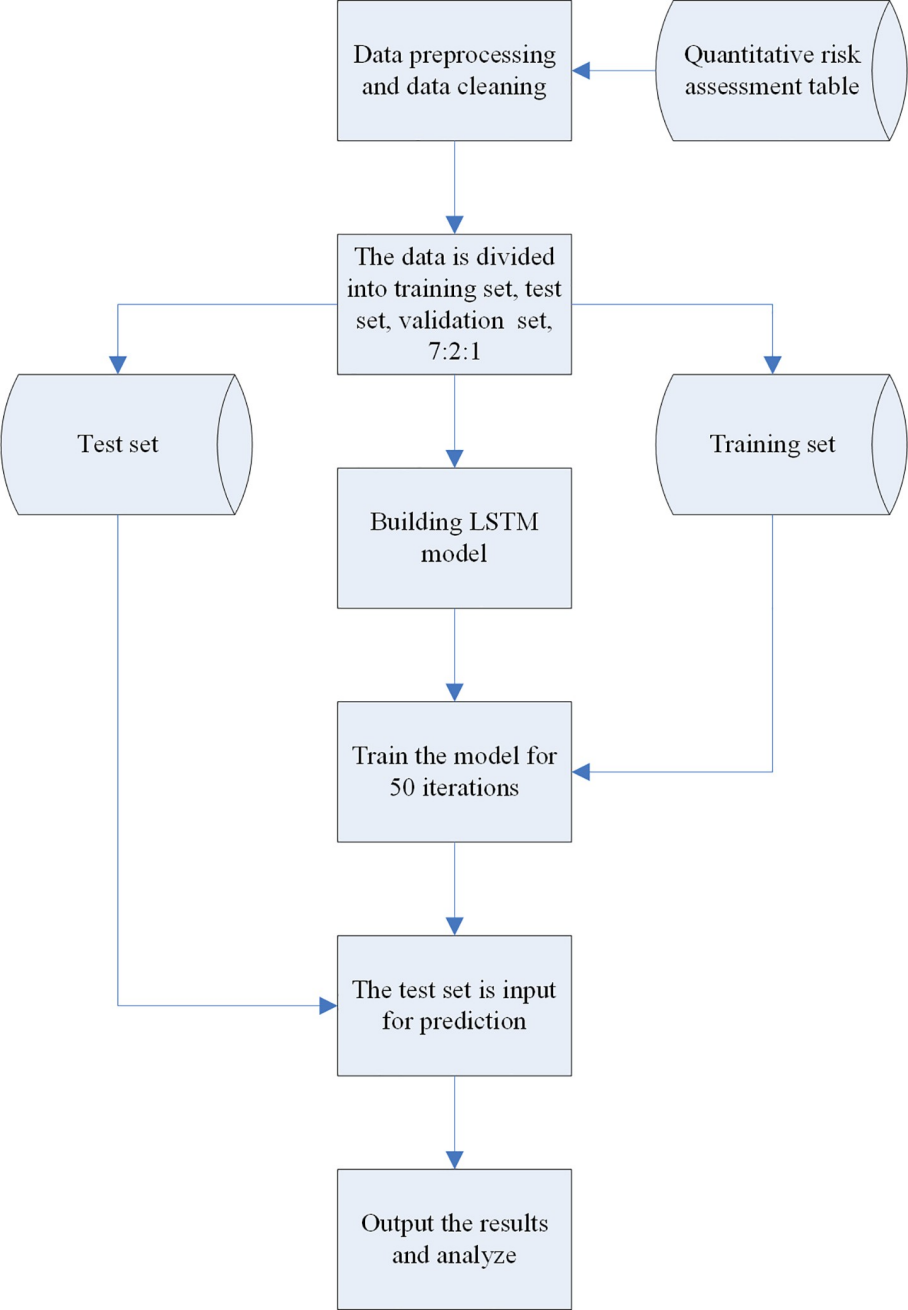
Intelligent risk assessment model diagram.

The airport security risk assessment model conducts risk analysis on four types of risk factors: airport facilities, airport staff, airport weather, and user activities. This article splits and matches the risk element information, and combines it with the risk assessment standard library of each profession to assign risk values to various risk assessment projects to obtain the total score. It uses LSTM algorithm for calculation and automatically outputs the risk levels of each project. This article can guide the preparation of various control measures, on-site monitoring, and scheduling of on-job positions based on different risk levels. This can ensure the safety, reliability, and resilience of airports, ensuring the safety and smooth operation of air traffic. Fig 2 is the intelligent risk assessment model.

**Fig 2** shows a flow chart of an intelligent risk assessment model. The process starts with data preprocessing, and goes through quantitative risk assessment and data cleaning. In the data preparation stage, the data is scientifically divided into training set, test set and validation set, with a ratio of 7:2:1. Subsequently, the LSTM model is used to build the model, and it is trained for 50 iterations to improve the accuracy of the model. Finally, the test set data is input into the trained model, and the prediction and output results are analyzed.

In this paper, the LSTM model has the ability to handle the long-term and short-term dependencies required for airport security risk assessment. The risk assessment data for airport security has the characteristics of time series, and the correlation of risk factors is directly related to historical data. LSTM can efficiently capture these long-term dependencies with its built-in memory gate mechanism. For the risk assessment task in this paper, the classic LSTM architecture is sufficient to cope with complex time series analysis, and no additional layer modification is required to improve the accuracy and efficiency of the model.

Taking the flight R of civil aviation in 2023 as an example, combined with the model and normal statistical methods, the fault Table 2 is output.

**Table 2:** R flight failure list.

Risk factor category	Specific risk factor	Probability of occurrence	Impact level	Preventive measures
Weather	Strong wind	0.05	Medium	Enhanced warning
	Thunderstorm	0.02	High	Emergency plans
	Rain	0.10	Medium	Adjust flight schedules
Airport facilities	Engine failure	0.03	High	Regular maintenance
	Landing gear failure	0.01	High	Key equipment monitoring
Staff	Pilot error	0.04	Medium	Enhanced training
	Security officer error	0.02	Medium	Strengthen operational norms
Others	Air traffic control	0.06	High	Optimized control processes
	Sudden passenger illness	0.01	Medium	Emergency medical facilities

In Table 2, in view of these risk factors, corresponding preventive measures are proposed, such as strengthening early warning, formulating emergency plans, regular maintenance and inspection, enhancing training, optimizing control processes and equipping first-aid facilities, so as to reduce flight delays and ensure passenger safety.

### 2.3 Long short term memory neural network

#### 2.3.1 RNN

In the RNN model, it solves the problem of restrictions on input and output data [19]. At present, there are many sequence data in reality, which usually have temporal correlations, that is, the network output at a certain time is not only related to the current input. It is also associated with a certain or several previous moments, and feedforward neural networks cannot handle this correlation. So a RNN has emerged, which has good memory and can make inferences based on previous memories. It can use memory ability to process input sequences of any time sequence, and associate previous memory with current input before outputting.

**Fig 3:**
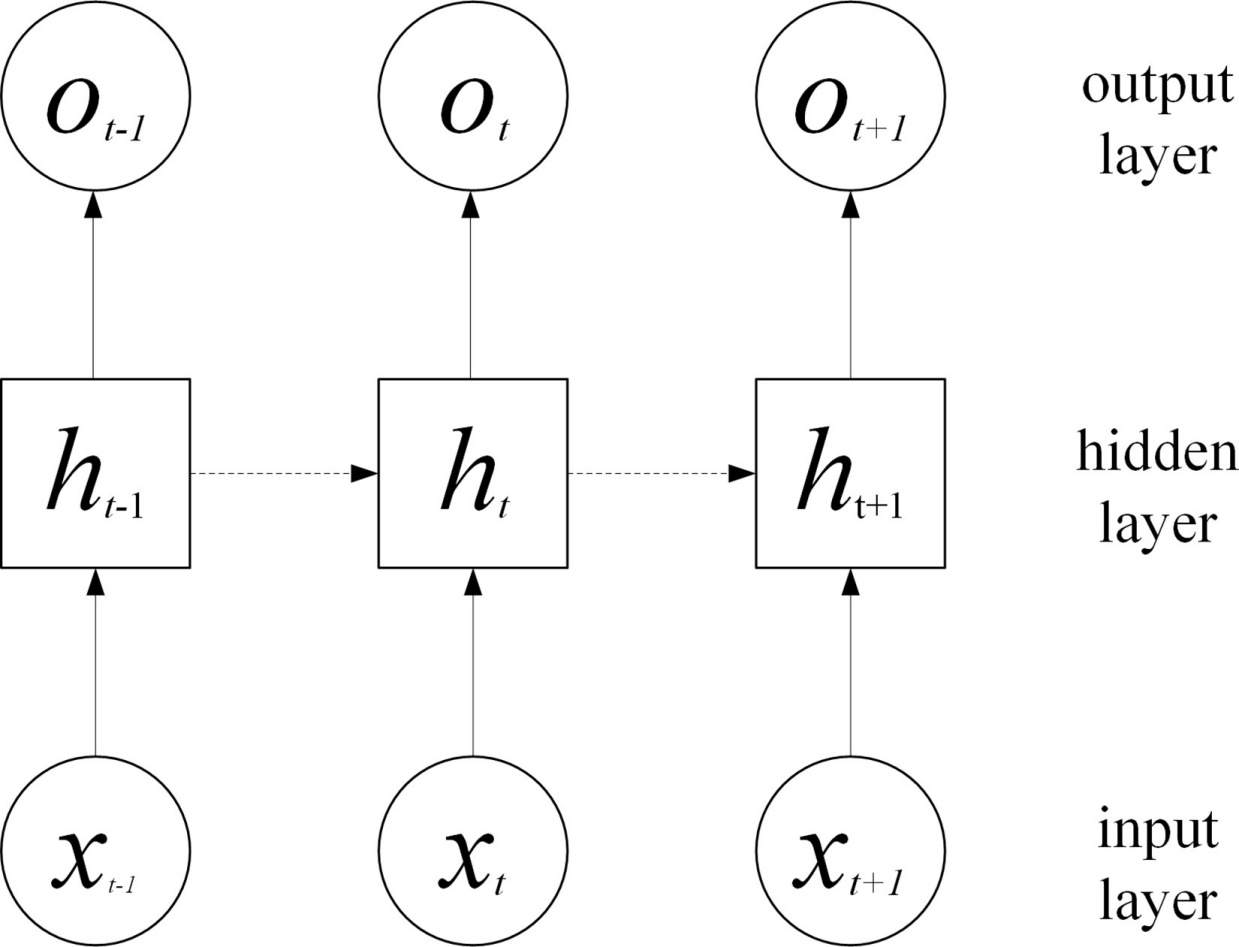
RNN network topology diagram.

Fig 3 is the network topology.

**Fig 3** is a diagram of the RNN network topology, which clearly shows the basic structure of the RNN network, including the input layer, hidden layer, and output layer (output layer). Each node in the figure represents a neuron, and the arrow indicates the direction of signal flow in the network. Through Fig 3, we can intuitively understand how the RNN network processes sequence data.

The forward propagation formula for RNN is:

$$\left\{ \begin{array}{c} h_{t}=f(W_{hh}h_{t-1}+W_{hx}x_{t}+b_{h})\\ o_{t+1}=W_{hy}h_{t}+b_{y} \end{array}\right.$$ (1)

h_t_ is the hidden state vector of the current time step t. F (·) is an activation function, usually using nonlinear functions such as tanh function or ReLU function. W_hh_ is the weight matrix of the hidden state vector h_t−1_. This loop structure enables RNN to process sequence data and capture time dependencies.

However, there are two common problems in RNN: gradient explosion and gradient disappearance [20]. When an RNN gradient explosion occurs, it can lead to numerical overflow and instability, making the model unable to converge and the training process unreliable, resulting in an increase in training time and a decrease in performance. When the RNN gradient disappears, the model may find it difficult to learn long-term dependencies, which hinders information transmission between long-distance time steps and reduces the accuracy and performance of the model.

To solve the gradient explosion and gradient vanishing problems in RNNs, we chose a variant called LSTM networks. An input gate decides which information from the current input should be stored in the cell state, a forgetting gate chooses to discard irrelevant information from the previous hidden state, and an output gate controls which part of the current cell state is output to the next layer.

In this paper, the encoding process of training data is to convert the text information related to flight delays into numerical form and input it into the LSTM model. The experiment first segmented and standardized the collected delay reason text to extract the risk factors in each flight record. Then, the risk factors were mapped into low-dimensional vectors using word embedding technology, and a time series was constructed based on the time characteristics of historical flight data. The risk indicators of the past 10 flights were used as input, so that the LSTM model can learn these time dependencies and predict the risk level of the next abnormal flight.

For the LSTM model, the input is a vector of length 4, containing four indicators: weather, facilities, staff, and user activities. The above indicators are used as input features of the model to capture various risk factors that affect airport security. The output of the model is the risk level, which represents the security risk level of the airport under specific input conditions. The goal of the LSTM model is to predict the risk level and provide an assessment of the airport’s security status based on these input data.

#### 2.3.2 Principle of LSTM model

**Fig 4:**
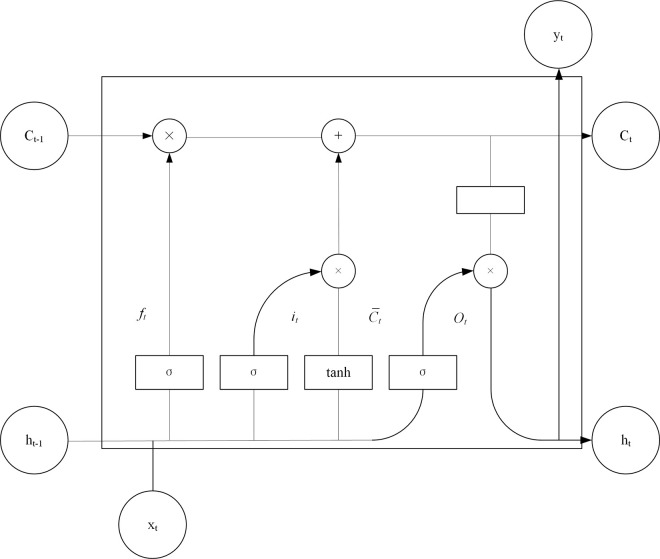
Structure diagram of LSTM cell model.

The integration scale at different moments can be dynamically changed, thus avoiding the problem of vanishing gradients or gradient inflation [21]. Compared with RNN, LSTM has a more complex hidden layer structure and can selectively remember or forget information. Through this gating mechanism and memory cell storage, LSTM can effectively handle long-term dependencies and perform well in sequence data modeling and prediction tasks. The following is Fig 4 of the LSTM cell model.

**Fig 4** is a structural diagram of an LSTM cell model, showing the main components of the model and their relationships. Among them, x_t_ represents the current time data input, c_t−1_ represents the memory state of the previous neuron, c_t_ represents the current neuron memory state, h_t−1_ represents the data output of the previous moment, y_t_ and h_t_ represent the data output of the current time. f_t_ represents forgetting gate, i_t_ represents input gate, o_t_ represents output gate, σ is sigmoid function, and tanh is activation function. “×” represents the multiplication of corresponding position elements within the matrix, and “+” represents the addition of corresponding position elements within the matrix.

The forward propagation process of the LSTM model is as follows [22]:

The function of sigmoid is to compress the output value to the range of [0,1], representing the proportion of information retained in the cell state of the previous time step. If the output value on a dimension approaches 0, it indicates that the corresponding information has been forgotten. If the output value is closer to 1, it indicates that the information should be retained more. The forgetting gate can filter and forget information, which is the key to solving the problem of RNN gradient disappearance and gradient explosion.

Specifically, the calculation process of the forgetting gate is as follows:

1. Input: the input sequence of the current time step is x_t_, and the output of the previous time step is h_t−1_, as well as the weight matrix and bias term.
2. Linear transformation: the input sequence can be linearly transformed with the hidden state of the previous time step to obtain a vector.
3. Sigmoid function: the result of a linear transformation can be activated through a sigmoid function to obtain the output of the forgetting gate.

$$f_{t}=\sigma (W_{hf}h_{t-1}+W_{xf}x_{t}+b_{f})$$ (2)

Among them, f_t_ represents the forgetting gate, W_xf_ represents the weight matrix of the input layer information flowing to the forgetting gate, W_hf_ represents the weight matrix of the hidden layer information flowing to the forgetting gate, b_f_ represents the bias of the forgetting gate, x_t_ represents the input at the current time, and h_t−1_ represents the output at the previous time.

In the forward propagation process of the LSTM model, input gates also play an important role in determining how much information is transmitted to the cell state from the current time step input. Specifically, the calculation process of the input gate is as follows:

1. Input: the input sequence x_t_ of the current time step, the output h_t−1_ of the previous time step, as well as the weight matrix and bias term.
2. Linear transformation: the input sequence can be linearly transformed with the hidden state of the previous time step to obtain a vector.
3. Sigmoid function: the result of a linear transformation can be activated through a sigmoid function to obtain the output of the input gate.
4. Result: The output of the input gate is dot product with the temporary cell state processed by a tanh function. A new memory state ${\bar{C}}_{t}$ containing candidate values can be output through a tanh layer, which can be added to the memory state to replace the previously selected forgotten information.

$$i_{t}=\sigma (W_{hi}h_{t-1}+W_{xi}x_{t}+b_{i})$$ (3)

$${\bar{C}}_{t}=tanh(W_{hc}h_{t-1}+W_{xc}x_{t}+b_{c})$$ (4)

Among them, W_xc_ represents the weight matrix of the input layer information flowing to the cell state, W_hc_ represents the weight matrix of the hidden layer information flowing to the cell state, b_c_ represents the bias of the cell state, x_t_ represents the input at the current time, and h_t−1_ represents the output at the previous time.

(5) Update cell state: firstly, perform dot product operation on the output i_t_ of the input gate and the temporary cell state processed by the tanh function. Combining the output f_t_ of the forgetting gate with the memory state ${\bar{C}}_{t}$, update C_t−1_ to C_t_, and its specific structure is shown in Formula (5):

$$C_{t}=f_{t}⊙C_{t-1}+i_{t}⊙{\bar{C}}_{t}$$ (5)

Output gates in the LSTM model:

The function of the output gate is to filter the current state of the neuron and output it, including the following two steps.

(1) Control the amount of information that the current input data x_t_ flows into the output gate through a sigmoid function.

Its specific structure is shown in Formula (6):

$$o_{t}=\sigma (W_{ho}h_{t-1}+W_{xo}x_{t}+b_{o})$$ (6)

(2) By using a tanh layer to transform the memory state C_t_ and combining its results with the output o_t_ of the previous step’s sigmoid, only the expected information can be output. The specific structure is shown in Formula (7):

$$h_{t}=o_{t}⊙tanh(C_{t})$$ (7)

In summary, the forward propagation process of the LSTM model includes calculation of input gates, calculation of forgetting gates, update of cell states, calculation of output gates, and update of hidden states. With these steps, LSTM models are designed to efficiently capture long-term dependencies in sequences and produce accurate outputs. This has led to LSTM models performing well in many sequence modeling tasks, such as natural language processing, speech recognition, and time series prediction.

For the LSTM model, hyperparameter optimization is performed by combining grid search and cross-validation. The experiment first defines a set of candidate values ​​for hyperparameters, then uses cross-validation to train and validate different hyperparameter combinations, and finally selects the hyperparameter settings that perform best on the validation set to ensure that the model can run under the optimal configuration and improve the overall performance of the model.

LSTM hyperparameter settings are shown in Table 3.

**Table 3:** LSTM hyperparameters.

Parameters	Value	Parameters	Value
Learning rate	0.001	Activation function	ReLU
Batch size	32	Dropout rate	0.2
Number of hidden units	128	Optimizer	Adam
Number of iterations	50	-	

## 3. LSTM risk assessment model

In the computer configuration for running the LSTM model, the hardware environment and software environment are as follows.

Hardware environment: Intel Core i7-9700K (eight cores), 32 GB DDR4 RAM, NVIDIA GeForce GTX 1080 (8 GB), 1 TB SSD.

Software environment: Windows 10 operating system, TensorFlow 2.6.0 framework, Python 3.8.

### 3.1 Data statistics

This article uses the 2022 security log dataset of a certain airline, which includes a total of 2190 logs. It contains a total of 7562 delayed flight information, including 25739 detailed reason texts. This article integrates these texts into statistics, and these reasons are divided into four basic categories based on risk factors: weather, user activities, airport facilities, and staff. The monthly proportion of various risk categories is shown in Table 4.

**Table 4:** Proportion of risk factors.

	Weather	Staff	Airport facilities	User activity
Month\quantity	14414 (56.0%)	1308 (5.1%)	2625 (10.2%)	7392 (28.7%)
January	1521	130	287	728
February	1203	98	178	623
March	998	82	182	545
April	1228	109	223	567
May	1342	103	214	575
June	1065	88	208	617
July	1600	125	246	587
August	1423	116	232	570
September	1032	95	214	598
October	1765	136	245	723
November	1152	101	176	635
December	1365	125	220	624

**Fig 5:**
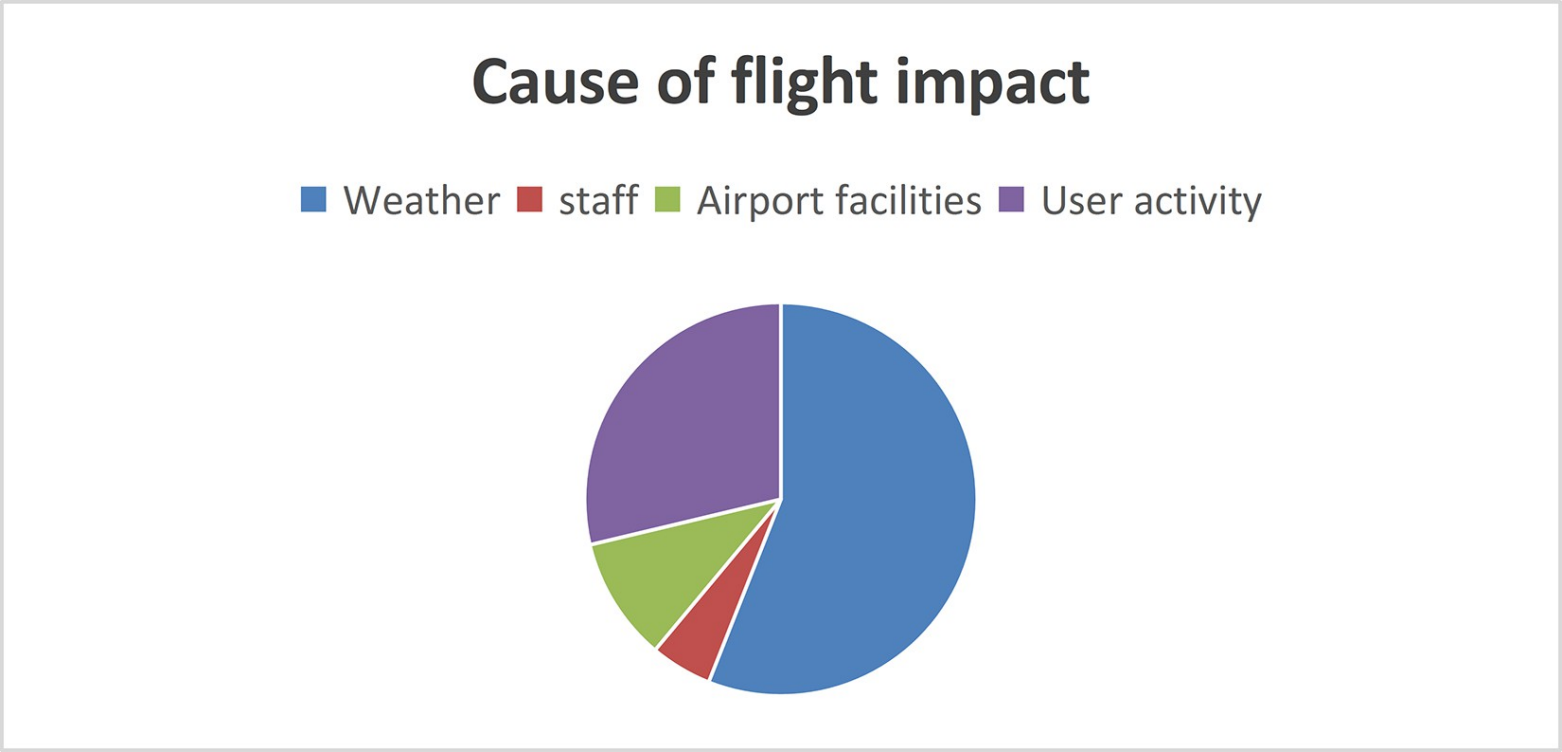
Proportion of reasons affecting flights.

The factors that affect flights are converted into a sector chart as shown in Fig 5.

Analysis shows that the vast majority of abnormal flights are mainly caused by weather and user activities. Accurately predicting aviation safety levels is of great significance for effective early warning and accident prevention. The complexity and incomplete understanding of the causal mechanisms and temporal characteristics of aviation crashes increase the operational costs of accurately predicting aviation security [23]. The impact of weather and user activities on flights is usually not fixed, some may be the main cause, while others may be secondary causes. Qualitative and quantitative analysis can be conducted on weather and user activities. This article divides different weight assignments based on the proportion of problem causes.

Based on expert experience and qualitative and quantitative analysis [24], this article divides weather into five levels, and a brief description is shown in Table 5. It is mainly determined by the degree and duration of weather impact. For example, heavy rain, which can affect aircraft visibility and last for a long time, is classified as a medium risk level, and may directly cause risks to aircraft due to thunderstorms. Because its duration is usually short, it is considered a medium to high level risk, while snowstorms can have a strong impact on aircraft visibility and balance ability, and their duration is generally long. In this case, it is considered a high level risk.

**Table 5:** Weather risk standards.

level of risk	Risk factor description	Value
Low risk	The intensity of weather influence is weak	1
Relatively low risk	The weather effect is moderate in intensity and short in duration	2
Moderate risk	The weather influence is moderate in intensity and long in duration	3
Relatively high risk	The weather influence is intense and short in duration	4
High risk	The weather effect is intense and lasts a long time	5

Similarly, user activities can be divided into three levels, as shown in Table 6. Users may carry prohibited items such as lighters, large capacity power banks, and oversized cosmetics with their personal belongings or luggage. These types of risky items are usually detected by security systems, and then airport staff need to temporarily detain these items. There may be a small number of users who may not comply with airport regulations, and at this point, the risk level would reach intermediate. When users begin to engage in attacks on staff, the airport risk becomes high, and security personnel need to be deployed to stop them and contact relevant departments to handle this matter. When customers carry a large amount of prohibited or illegally smuggled items, it would also be judged as a high risk.

**Table 6:** User activity risk standards table.

level of risk	Risk factor description	Value
Low risk	The user is carrying a small number of prohibited items	1
Moderate risk	User conflicts with airport personnel	2
High risk	The user has illegal or above behavior	3

The two major types of risk factors, airport facilities and airport staff, usually have fewer problems, so their weight values are reduced, that is, if problems occur, they are set to 1, otherwise 0. Finally, these assignments are simply weighted to obtain the airport’s risk indicators, and an airport risk level table is divided. The description of each risk level is shown in Table 7.

**Table 7:** Airport risk levels.

RISK GRADE	RISK INDICATOR	LEVEL OF RISK	DATE REMARK
**1**	0–1	Low risk	Basically no effect
**2**	2–3	Relatively low risk	There are a small number of risk factors, which may affect flights and need to be dealt with as soon as possible
**3**	4–5	Moderate risk	There are risk factors that may affect flights and need to be handled urgently
**4**	6	Relatively high risk	There are a lot of risk factors, and flight operation needs to wait until the risk factors decline
**5**	7–10	High risk	The airport should be temporarily closed

### 3.2 Data preprocessing

After completing the data statistics, the text information is transformed into clear data based on risk factors, where the time series is constructed using the time information of abnormal flights as a sequence. This study constructed a dataset containing flight information for 2358 flights, and the processed data is shown in Table 8. Based on the LSTM algorithm, this article predicts the risk level of the next abnormal flight using the risk indicators of the past 10 flights. Complex and numerous risk factors can be simplified into risk indicators through qualitative and quantitative methods. This decreases the complexity of the model and increases its robustness.

**Table 8:** Airport security risk data.

Airport weather risk indicators	Airport facility risk indicators	Airport staff risk indicators	User Activity Risk indicators	Risk grade	Flight departure time
4	1	0	1	4	2022/1/1
4	2	1	1	5	2022/1/2
0	0	0	1	1	2022/1/3
4	1	0	1	4	2022/1/4
3	0	0	0	2	2022/1/5
3	0	0	2	3	2022/1/6
5	2	0	2	5	2022/1/7
3	0	0	2	3	2022/1/8
. . .	. . .	. . .	. . .	. . .	. . .
4	1	0	0	3	2022/12/28
2	1	0	1	3	2022/12/29
1	0	0	1	2	2022/12/30
0	1	0	0	1	2022/12/31

The dataset can be divided into input and output, and the next occurrence of airport risk can be predicted through 10 consecutive airport risks in a certain period of time, that is, the input time series length of the LSTM model is 10, and the output time series length is 1. In terms of feature selection, since the input and output only have a risk level, the number of features is 1.

The irregularity of the risk level data may lead to inefficient learning, low accuracy and convergence difficulties of the LSTM model. In order to improve the accuracy of the model, the denoising of the data using the Hanning window factor can be considered to decrease the learning difficulty of the LSTM model [25].


$$w(n)=0.5-0.5\;\cos\left(\frac{2\pi n}{M-1}\right)$$ (8)

To improve the efficiency of model training and prevent overfitting, the standardized scaler method can be used to further process the data before model training. The normalized definition of the standardized scaler is shown in Formula (9). *X*′ is the converted data, *X* is the raw data, *μ* is the mean of all sample data, and *σ* is the standard deviation of all sample data [26].


$$X\prime =\frac{X-\mu }{\sigma }$$ (9)

LSTM model training and prediction analysis were carried out on the risk value after data discretization and de-noising.

### 3.3 Model performance verification

**Fig 6:**
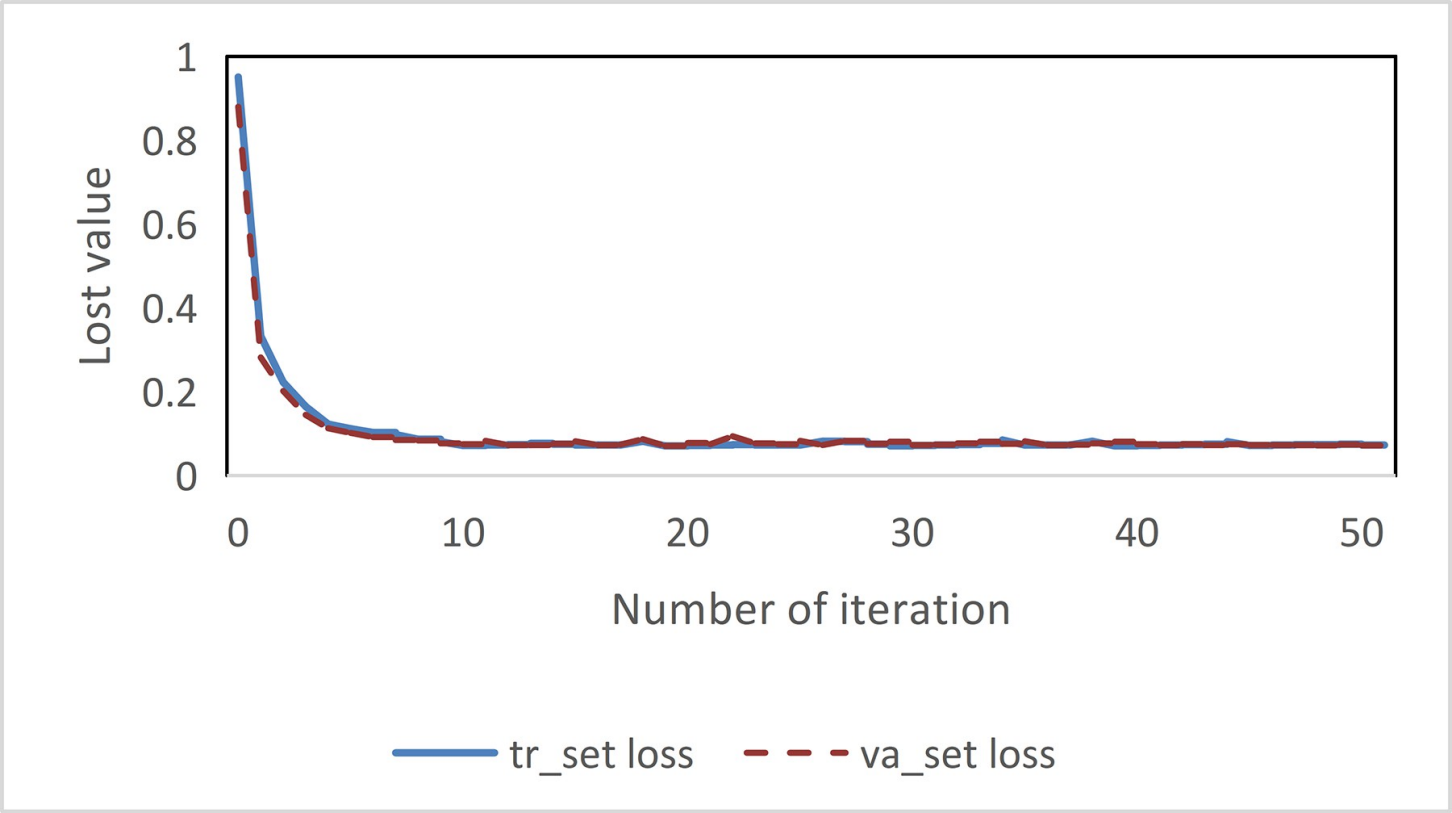
LSTM model training stability verification.

In Fig 6, the training set Loss tr_set Loss and verification set loss va_set Loss in the trained LSTM model are plotted as parameters of the evaluation model. AS the number of iterations increase, the values of tr_set Loss and va_set Loss decrease and converge steadily to 0.073, and the model as a whole reaches a stable state.

**Fig 7:**
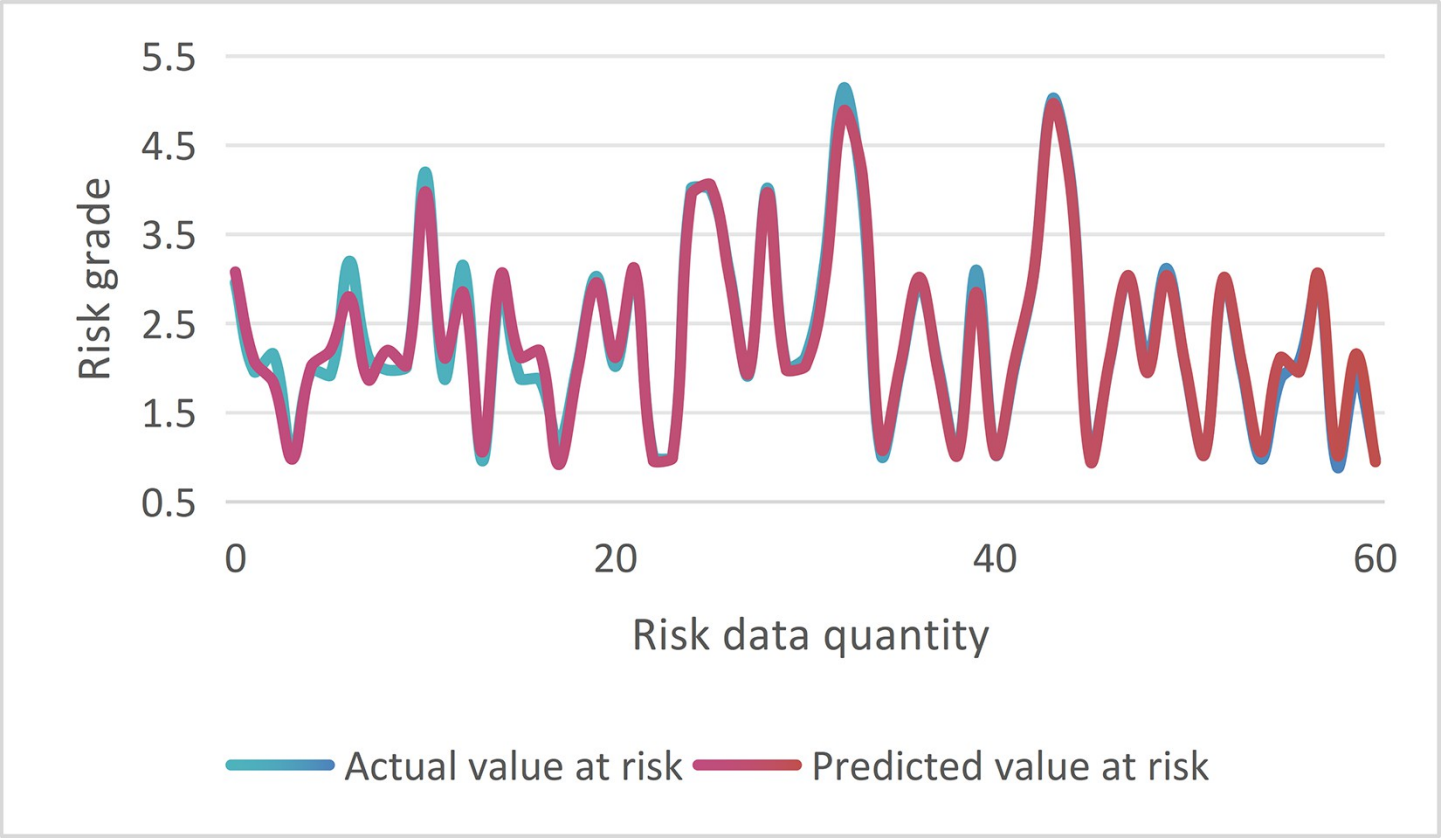
LSTM model training accuracy verification.

The experiment input 60 test set risk data into the trained LSTM model and compared them with the actual values. As shown in Fig 7, it can be seen that the trends of the two are highly consistent, which verifies the good fitting ability of the model.

The initial weights of ML networks in the initial few training sessions are random, and there may be errors between the final trained model and the predicted results. To verify the robustness of the model, this article conducted 10 training sessions on the LSTM model. It uses the root mean square error (RMSE) and coefficient of determination (R^2^) between the predicted values obtained after model training and the real labels as indicators to evaluate model performance. The closer RMSE is to 0, the more accurate the LSTM model is. The closer R^2^ is to 1, the more accurate the LSTM model is.


$$RMSE=\sqrt{\frac{\sum_{i=1}^{n}{\left(y_{i}-\hat{y_{i}}\right)}^{2}}{n}}$$ (10)

$$R^{2}=1-\frac{\sum_{i=1}^{n}{\left(y_{i}-\hat{y_{i}}\right)}^{2}}{\sum_{i=1}^{n}{\left(y_{i}-\bar{y_{i}}\right)}^{2}}$$ (11)

Among them, y_i_ represents the true risk value, $\hat{y_{i}}$ represents the predicted risk value, and $\bar{y_{i}}$ represents the average risk value.

In Table 9, each column represents a different round of model training.

**Table 9:** Standard error and determinable coefficients of model training 10 times.

	1	2	3	4	5	6	7	8	9	10
RMSE	0.173	0.175	0.179	0.171	0.172	0.171	0.173	0.172	0.178	0.174
R^2^	0.902	0.907	0.904	0.908	0.906	0.904	0.905	0.904	0.903	0.904

The comparison outcomes are shown in Table 9. After 10 trainings, the standard error of the model is less than 0.18, and the decision coefficients are all greater than 0.9. Therefore, it can be summarized that the model has a high forecasting accuracy. The fluctuation amplitude of the indicators of standard error and determinable coefficient is relatively small, indicating that the predicted results of the model have strong stability and good robustness.

### 3.4 Prediction results

This article trains and tests the model by obtaining the annual aviation fault log data of a civil aviation airport in 2022. After preprocessing the log data of this study, the paper chose to use random sampling to extract the risk values of 10 flights for prediction, and the results are shown in Table 10. Through model validation, the predicted results of the airport safety risk assessment model using the LSTM algorithm are close to the true values. It can be considered that the model has high prediction accuracy.

**Table 10:** LSTM model validation results.

Actual risk indicators	Actual risk grade	Predicted value at risk	Date
4	3	2.97	2022/1/1
3	2	2.13	2022/1/8
6	4	4.02	2022/2/12
1	1	0.85	2022/3/21
5	3	3.18	2022/4/7
2	2	1.88	2022/6/15
7	5	4.62	2022/7/20
3	2	2.08	2022/8/23
2	2	1.82	2022/10/6
1	1	0.92	2022/12/1

To enhance comparability, continue to select other established methods: ARIMA model, decision tree model, naive Bayes model, support vector machine model for prediction comparison. The details are shown in Table 11 below.

**Table 11:** Comparison of predictions with other established methods.

Model	Actual risk grade	Predicted value at risk	Accuracy metrics	Comparison notes
LSTM	4	2.97	MAE: 0.25, RMSE: 0.35, R^2^: 0.90	High accuracy
ARIMA	3	2.13	MAE: 0.28, RMSE: 0.41, R^2^: 0.80	Moderate accuracy
Decision Tree	6	4.02	MAE: 0.30, RMSE: 0.45, R^2^: 0.75	Lower accuracy
Naive Bayes	1	0.85	MAE: 0.27, RMSE: 0.40, R^2^: 0.85	Moderate accuracy
SVM	5	3.18	MAE: 0.29, RMSE: 0.43, R^2^: 0.82	Moderate accuracy

Table 11 shows the comparison between the flight safety risk values predicted by the LSTM-based model in this paper and four other traditional models. The LSTM model forecasts risk values close to the actual risk level with high accuracy, with an MAE of 0.25, an RMSE of 0.35, and an R^2^ of 0.90. Overall, the LSTM model performs the best in predicting aviation safety risks.

The intuitive reason for predicting the risk level for a given day in relation to various risk indicators and the risk levels of the previous 10 days is mainly the temporal correlation between risk indicators and historical data. Airport safety risk assessment relies on a variety of factors, including weather, user activity, airport facilities and staff performance, which have a cumulative effect on flight safety over a certain time frame. This paper analyzes the risk levels of the past 10 days to reveal potential trends and cyclical patterns, and more accurately estimate the upcoming risk level.

The comparison results of the experimental model LSTM with other literature are shown in Table 12.

**Table 12:** Comparison results with other literature.

Model	MAE	RMSE	R^2^
LSTM	0.25	0.35	0.9
Fuzzy comprehensive evaluation model [27]	0.3	0.4	0.85
TF-IDF and TextRank [28]	0.28	0.38	0.88
LSTM-BP [29]	0.22	0.3	0.92

In Table 12, we can see that the LSTM model has a high prediction accuracy, with MAE reaching 0.25, RMSE 0.35, and R^2^ 0.90. Compared with the fuzzy comprehensive evaluation model, TF-IDF and TextRank are better. The LSTM-BP model performs best, with MAE of only 0.22, RMSE of 0.30, and R^2^ 0.92. The performance of the LSTM model in this experiment is not much different from that of the LSTM-BP model in the literature, which shows that the LSTM in this experiment is feasible.

## 4. Conclusions

Risk assessment and prevention are an important aspect of ensuring airport safety. By predicting potential risks and preparing in advance for the actual arrival of risks, it is important to ensure the safety of the airport. This is of great significance for airport safety. This study conducted a qualitative and quantitative analysis of airport safety risk factors, and developed a risk quantification table to convert uncertain factors into quantifiable data, which provides a scientific basis for risk assessment. A risk assessment and prediction model based on LSTM algorithm has been constructed. By training and predicting the LSTM model, this article verified the stability and robustness of the LSTM model. It compared the predicted data of the model with the actual data, and the two data had a high degree of fitting. This shows that the airport security risk assessment model based on LSTM is helpful to quickly identify security risks, improve work efficiency, prevent security risks, and ensure airport safety. Airport managers can make corresponding decision support according to the risk assessment results provided by the model, optimize resource allocation, enhance real-time monitoring ability, and improve the overall security level. The data set selected in this paper has a small time span and small scale. In the future, the scope of the data set can be expanded, more regional airport information and longer time span data can be selected to improve the universality and accuracy of the model, and bidirectional Long Short-Term Memory (Bi-LSTM) network can also be considered to continue to improve the modeling ability.

## Supporting information

S1 Data (CSV)
